# The need for race-specific reference equations for pulmonary diffusing capacity for nitric oxide

**DOI:** 10.1186/s12890-021-01591-7

**Published:** 2021-07-13

**Authors:** Gerald Stanley Zavorsky, Ahmad Saleh Almamary, Mobarak Khalid Alqahtani, Shi Huh Samuel Shan, Douglas Shawn Gardenhire

**Affiliations:** 1 Pulmonary Services Department, University of California, Davis, Medical Center, 2315 Stockton Boulevard, Room 5703, Sacramento, CA 95817 USA; 2 Faculty of Medicine, National Heart and Lung Institute, Imperial College London, London, UK; 3 Institute of Cardiovascular Sciences, University of Birmingham, Birmingham, UK; 4 Department of Respiratory Therapy, Georgia State University, Atlanta, GA USA

**Keywords:** Ethnicity, Prediction equations, Lung function, False positives, Carbon monoxide

## Abstract

**Background:**

Few reference equations exist for healthy adults of various races for pulmonary diffusing capacity for nitric oxide (DLNO). The purpose of this study was to collect pilot data to demonstrate that race-specific reference equations are needed for DLNO.

**Methods:**

African Americans (blacks) were chosen as the comparative racial group. In 2016, a total of 59 healthy black subjects (27 males and 32 females) were recruited to perform a full battery of pulmonary function tests. In the development of DLNO reference equations, a white reference sample (randomly drawn from a population) matched to the black sample for sex, age, and height was used. Multiple linear regression equations for DLNO, alveolar volume (VA), and pulmonary diffusing capacity for carbon monoxide (DLCO) using a 5–6 s breath-hold were developed.

**Results:**

Our models demonstrated that sex, age^2^, race, and height explained 71% of the variance in DLNO and DLCO, with race accounting for approximately 5–10% of the total variance. After normalizing for sex, age^2^, and height, blacks had a 12.4 and 3.9 mL/min/mmHg lower DLNO and DLCO, respectively, compared to whites. The lower diffusing capacity values in blacks are due, in part, to their 0.6 L lower VA (controlling for sex and height).

**Conclusion:**

The results of this pilot data reveal small but important and statistically significant racial differences in DLNO and DLCO in adults. Future reference equations should account for racial differences. If these differences are not accounted for, then the risk of falsely diagnosing lung disease increase in blacks when using reference equations for whites.

**Supplementary Information:**

The online version contains supplementary material available at 10.1186/s12890-021-01591-7.

## Introduction

Pulmonary function tests (PFTs) are essential tools for modern clinical respiratory function assessment of. They are used in the evaluation of patients with respiratory symptoms and for guiding the management of diagnosed lung disease. Many patients get misdiagnosed and are improperly treated without the guidance of PFTs. One such PFT, pulmonary diffusing capacity for carbon monoxide (DLCO), is a single-breath technique using a 10-s breath-hold maneuver that was clinically established in 1957 [1]. Since 1957, the single-breath DLCO test has become the clinical standard to assess gas transfer through the lung. Prediction equations for DLCO were developed to decipher normal from abnormal gas transfer through the lung. These equations have been created for adults [2–8] and children [9–11], with a wide range of racial backgrounds [11–20].

While carbon monoxide (CO) has been the transfer gas of choice for the measurement of diffusing capacity, the use of nitric oxide (NO) as a potential alternative originated much later by separate and independent research teams [21]. The first two abstracts on DLNO originated from the United Kingdom in the 1980s [22, 23]. These abstracts resulted in the first publications of DLNO [24, 25].

There is evidence to suggest that NO could be a better transfer gas compared to CO, or at least, used alongside CO [26]. The chief barrier to CO uptake (~ 70–80%) resides within the red cell (i.e. red cell resistance) while ~ 25% is located in the alveolar membrane (See Fig. 1 elsewhere [27]). In contrast, the main barrier for NO uptake resides between the alveolar and red blood cell membranes (~ 60%) (i.e. membrane resistance) [28]. This advantage gives DLNO a better representation of gas transfer through the alveolar-capillary membrane compared to DLCO. Unlike DLCO, DLNO is relatively unaffected by changes in hemoglobin concentration [29], carboxyhemoglobin concentration [30], alveolar oxygen pressure (P_A_O_2_), or inspired oxygen concentration [31, 32]. Furthermore, DLNO is more affected by lung volume compared to DLCO, which makes the KNO (mathematically, DLNO divided by alveolar volume) a better measure than KCO (mathematically, DLCO divided by alveolar volume) in those with restrictive lung disease [33]. Finally, the sensitivity in detecting cardiopulmonary disease may be improved using DLNO compared to DLCO [26]. These examples demonstrate there is evidence that a DLNO test can be technically and physiologically superior to a DLCO test [26].

Few prediction equations exist for single-breath DLNO in adults [34–38] and children [39–41] (see Additional file 1: Table S1). In addition to measurements of DLNO, DLCO prediction equations have also been developed (Additional file 1: Table S2). In 2017, a European Respiratory Society Task Force (ERS) published reference equations for DLNO [27] based on combined data from three studies evaluating nearly 500 white subjects [34–36]. However, no prediction equations for DLNO have been developed in the African-American population (herein known as the black population).

Ethnic/racial differences exist in certain aspects of lung function. For example, 15% of the variability in vital capacity is accounted for by race/ethnicity [42]. Vital capacity (i.e., lung volume) is about 15% lower in blacks compared to age, height, and sex-matched whites [13, 43]. Moreover, the DLCO is also lower in blacks compared to matched whites [13, 19]. Specifically, DLCO is lower by ~ 2 (~ 6%) mL/min/mmHg in black, age and height-matched males compared to matched white males [19]. DLCO is also lower by ~ 5 (~ 15%) mL/min/mmHg lower in black, age and height matched females compared to matched white females [19]. The difference in DLCO between blacks and whites stems from differences in alveolar volume and hemoglobin concentration as there is a ~ 6% larger hemoglobin concentration in whites compared to blacks [19, 44]. Although DLNO is minimally affected by hemoglobin concentration [29], the development of DLNO prediction equations for blacks is justified given the differences in lung volumes between the two different ethnic/racial groups. One study suggested that blacks originating and living Sub-Sarahan Africa have lower DLNO values compared to whites [45].

Racial differences in lung function could result in significant public health consequences if improper reference equations are used. The use of incorrect reference equations could result in the overdiagnosis of lung disease in the black population. Misdiagnosis of lung disease could result in increased patient stress and inadvertent use health care resources, resulting in a higher cost and potential harm forpatients with a false positive diagnosis. We therefore sought to demonstrate that reference equations are specifically needed for DLNO in the adult black population in the United States by sampling a black university population in Atlanta, GA. To date, there are no reference equations developed for DLNO in the African American population. As such, this study’s primary aim was to determine if racial differences exist for DLNO. Pulmonary diffusing capacity in the black population was compared against sex, age, and height-matched white adults used in the 2017 ERS Technical Standards document for DLNO [27]. Moreover, since breath-hold time can alter DLCO, alveolar volume (VA), and logarithmic change in CO concentration per unit time and unit pressure (KCO) [46], a secondary aim was to evaluate 10 s and 5 s breath-hold times on those variables as secondary outcomes in the black population.

## Methodology

Healthy black male and female non-smoking adults from Georgia State University (GSU) were recruited to participate in one testing session involving the measurement of various lung parameters, including DLNO and DLCO. Subjects were selected based on responses to a flyer that was posted around campus. This was a descriptive observational study performed in conjunction with another study examining the six-minute walk test in this same ethnic/racial group. The study was approved by the Georgia State University ethics board (IRB #H16120, Reference # 335,588). Informed consent was obtained prior to study participation. The inclusion and exclusion criteria were as follows:

### Inclusion criteria

- A representative sample of the university African American population, who are non-smoking and non-pregnant
- Individuals ≥ 18 years of age, with a body mass index (BMI) ranging from 17.0 to 34.9 kg/m^2^. Non-smoking was defined as never smoked or quit smoking > 6 months previously.
- Subjects did not have cardiopulmonary disease or signs/symptoms suggestive of cardiopulmonary disease [47].

### Exclusion criteria

- Those that did not fit the inclusion criteria above, and:
- Those that have chest or abdominal pain or any cause, oral or facial pain exacerbated by a mouthpiece, stress incontinence, dementia, or in a state of confusion [48].

### Procedures

Subjects signed an informed consent form to participate in the study. Procedures lasted approximately 1.75 h in total per subject. Subjects filled out a questionnaire assessing demographic data including date of birth, sex, a physical activity readiness questionnaire (PAR-Q), and a health questionnaire. Height, weight, waist, and hip circumference were measured before the pulmonary function tests. Heart rate was measured via a POLAR A300 heart rate monitor (Polar Electro Oy, Kempele, Finland) during the PFTs. Average heart rate was recorded during these PFTs (i.e., 20–30 min) and used for data analyses.

Pulmonary function tests were performed in half of the subjects the following order: (a) slow vital capacity (SVC), (b) Spirometry, (c) 5 s breath-hold NO–CO double diffusion measurement (DLNO_5s_, DLCO_5s_), (d) total lung capacity (TLC), and (e) DLCO (10 s breath-hold, DLCO_10s_). In the other half, the order of (c) and (e) were reversed. As such, the first participant that was scheduled to come to the lab for testing performed tests a-e in sequence. Then the next subject that was scheduled for testing completed tests a, b, e, d, c in that order. The order of testing alternated back and forth until the end of the recruitment.

The procedures, rules, and evidence of established safety for conducting these lung function tests has been previously described [49–53]. The diffusing capacity test involved subjects inspiring approximately 4–6 L of a standard diffusion gas mixture that is used for diagnostic purposes, including a small amount of NO (i.e., 0.3% CO, 21% O_2_, 10% He, 40–60 ppm NO, Balance N_2_). This gas mixture was inhaled once to total lung capacity, held for 5–10 s, and then and then exhaled.

The Hyp'Air lung diffusion system (Medisoft Inc., Sorinnes, Belgium) was used for the assessment of DLNO, VA, KNO, DLCO, and KCO using the 5 s breath-hold maneuver. Nitric oxide and CO electrochemical cells were used to measure inhaled and exhaled NO and CO concentrations. Insofar as the resolution of the NO electrochemical cell is in the ppm range, 5s breath-hold maneuvers were used to prevent exhaled NO from being in the ppb range. The Medisoft Body Plethysmograph (BODYBOX 5500^®^ Series, Medisoft Inc., Sorinnes, Belgium) was used for the assessment of spirometry, TLC, DLCO (10 s breath-hold maneuver), VA (10 s breath-hold maneuver), and KCO (10 s breath-hold maneuver). The best values for spirometry were reported [49], and the mean value for DLNO and DLCO was reported when the two highest DLCO values varied by not more than 3 mL/min/mmHg, and the two highest DLNO values did not vary by more than 17 mL/min/mmHg [27]. For the measurement of TLC from the body box, three values for FRC that agreed within 5% were obtained, and the mean was reported [51].

Subjects were paid $30 for their participation. The funding for this study came from the Jerome M. Sullivan Research Fund from the American Respiratory Care Foundation.

### Calculation of DLNO, DLCO

Recommended guidelines from the ATS and ERS were used to calculate DLNO_5s_, DLCO_5s_, and DLCO_10s_ [27, 53]. Standardized hemoglobin concentrations were used for men (14.6 g/dL) and women (13.4 g/dL), respectively, was used, and a P_A_O_2_ of 100 mmHg was also used as recommended by the ERS Task Force [27].

### Statistical analyses

Spirometry values (FVC, FEV_1_, FEV_1_/FVC ratio) were compared to predicted values for the black population [43]. Total lung capacity was compared to the predicted values for a white population [3]. The mean predicted values were compared to the mean measured values via a paired *t-*test. When developing reference equations, the number of subjects should be sufficiently large because the larger the sample, the more likely it will be to represent the population. It is known that there is a direct relationship between the correlation and the ratio of the number of independent variables (IVs) in the model (*k*) to the number of participants in the model (*n*), such that (*k* − 1) ÷ (*n* − 1) [54]. Thus, if a study has 40 participants and 30 IVs, the R^2^ would be 0.74 based on chance alone, and the results would be meaningless; therefore, it is recommended that there be at least a 10:1 participant to variable ratio to avoid this error [54]. In this case, where there are nine potential predictors for DLNO (age, age^2^, age^3^, sex, weight, height, height^2^, height^3^, race), at least 90 subjects would be needed.

A multiple linear regression model using the forward procedure was conducted to determine which IVs (age, age^2^, the interaction term age^3^, sex, weight, height, height^2^, the interaction height^3^, race) were predictors of DLNO_5s_ (first dependent variable), DLCO_5s_ (second dependent variable), and alveolar volume (third main dependent variable). Forward selection first determined the bivariate correlations among all IVs and the dependent variable. The procedure then determined which IV is most highly correlated with the dependent variable and chose it as the first significant predictor, and it remained in the equation. The next variable entered in the analysis was the independent variable that contributed most to the dependent variable after partialling out the effects of the first independent variable. This was measured by the increase in R^2^ due to the second variable. Once this IV is chosen, it remained in the equation. This procedure continued until an IV stopped making contributions to the dependent variable [55]. Any predictor variable that was statistically significant (*p* < 0.05) was initially kept in the model. However, it was also important to note efficient regression equations were developed without including everything across the board. For example, if age correlated nearly as close as age∙age^2^, age was used instead for simplicity. Since regression was very sensitive to extreme cases, outliers were removed. Any data point that demonstrated a standard deviation of the residuals ≥ 3.5 was eliminated. Linearity was analyzed by creating a scatterplot matrix of variables. Another plot was created between the standardized residuals (y-axis) and standardized predicts (x-axis) to see if the values were consistently spread out, which would indicate normality and homoscedasticity. When multicollinearity was examined, the variance inflation factor (VIF) was used to see whether there was a strong association between the independent and dependent variables. All independent variables in the model had a VIF near one [56]. If any predictor had a VIF of more than five, it was removed from the model [57]. A Durbin–Watson test was performed to test if serial errors were correlated [58, 59]. The range was 0–4: a value of nearly two indicates non-autocorrelation, a value close to zero indicates a positive autocorrelation, and a value close to 4 indicates a negative autocorrelation [60].

The number of male and female black subjects recruited was then matched with the exact same number of male and female white subjects, randomly selected from a sex, age, and height matched dataset used to generate reference equations for DLNO [27]. These were historical white control subjects obtained from the 2017 ERS Taskforce publication on the standardization of DLNO [27]. To examine differences in DLCO, VA and KCO between 10 and 5 s breath-hold maneuvers, simple linear regressions, paired *t*-tests, and Bland–Altman plots [61] were used.

A Type I probability level of 0.05 was used. The statistical software utilized was IBM SPSS Statistics Version 26.0, IBM Corporation, Chicago, IL.

## Results

Sixty adult black subjects were recruited from GSU over a period of 6 months in 2016 (Table 1). One subject had missing data and was therefore removed from the analysis. The remaining fifty-nine subjects (32 females, 27 males) completed all tests. The subjects ranged from 18 to 67 years of age, and the mean resting heart rate was 80 (SD 10) beats/min. Two African American subjects (3% of the black sample) were ex-smokers, with each having a previous exposure of two-pack years. One of the subjects that previously smoked quit 1.5 years before the start of the study, and the other subject quit 1 year before study commencement. The remining African American subjects were never smokers.

**Table 1:** Anthropometric characteristics

	2017 ERS technical standards (white subjects)	Current study African Americans (black subjects)
*Males (n* = *27 per group)*		
Age (years)	30.1 (6.0)	28.0 (9.5)
Height (cm)	179 (8)	176 (7)
Weight (kg)	76.2 (10)	78.6 (11.1)
BMI (kg/m^2^)	23.7 (2.2)	25.3 (2.9)*
*Females (n* = *32 per group)*		
Age (years)	31.8 (8.4)	31.9 (13.6)
Height (cm)	166 (6)	163 (8)
Weight (kg)	59.8 (8.4)	68.2 (13.9)*
BMI (kg/m^2^)	21.6 (2.4)	25.7 (5.3)*

^*^p < 0.05 between white and black subjects

A white reference sample of exactly 32 females and 27 males used in the development of DLNO_5s_ reference equations was randomly drawn from a population matched to the black sample for sex, age, and height. All these subjects were never smokers. The statistics program SPSS randomly sampled the white reference subjects from the dataset used elsewhere [27]. There were no differences in mean age or height between whites and blacks (Table 1). More detailed anthropometric characteristics of the black subjects are presented in Additional file 1: Table S3.

The mean FVC and FEV_1_ in the black subjects were statistically larger compared to the percent predicted for race, age, height and sex (Additional file 1: Table S4). However, interestingly, TLC was ~ 100% predicted in the black subjects (Additional file 1: Table S4), which was a prediction equation created for white subjects.

Due to the likelihood of inadequate gas penetration in the lung with a 5 s breath-hold maneuver compared to a 10 s breath-hold maneuver, DLCO_10s_ was compared to the DLCO_5s_ in the black subjects. There was an ~ 8% coefficient of variation between DLCO_10s_ and DLCO_5s,_ but there was no mean difference between the two breath-hold times (*p* > 0.8) (Additional file 1: Table S5, Figure S3). About 80% of the variance DLCO_10s_ was accounted for by DLCO_5s_ (Additional file 1: Figure S1). Furthermore, there was a 6% coefficient of variation between VA_10s_ and VA_5s_, and a + 0.15 L (SD 0.40) higher mean value (i.e., 3% higher) with the 5 s breath-hold compared to the 10 s breath-hold maneuver in the black subjects (*p* < 0.01) (Additional file 1: Table S5, Figure S4). Approximately 88% of the variance in VA_10s_ was shared by VA_5s_ (Additional file 1: Figure S2). There was an ~ 6% coefficient of variation between KCO_10s_ and KCO_5s_, with KCO_10s_ showing a 0.16 mL/min/mmHg/L larger value compared to KCO_5s_ (p < 0.01, Additional file 1: Figure S3 and S6). About 74% of the variance KCO_10s_ was accounted for by KCO_5s_ (Additional file 1: Figure S1).

The DLNO_5s_ ranged from 71 to 205 mL/min/mmHg in the black subjects (Additional file 1: Table S6). The specific concentrations of inspired and expired gases as well as the actual breath-hold times are found in Additional file 1: Table S7. The DLNO_5s_ was about 5 × larger than the DLCO_5s_ (Additional file 1: Table S6) with the regression equation being DLNO_5s_ = 4.65∙(DLCO_5s_) + 10.94, R^2^ = 0.93, SEE = 9.2 mL/min/mmHg, *p* < 0.01. The 95% CI for the slope between DLNO_5s_ and DLCO_5s_ was 4.30–5.00.

Heart rate at rest was negatively associated with DLNO_5s_ and DLCO_5s_ in the black subjects (Additional file 1: Figure S7). When the effects of age were partialled out, there was an 18–21% shared variance between heart rate and diffusing capacity.

Multiple linear regression results are presented in Tables 2, 3 and 4. Approximately 72% of the variance in DLNO_5s_ was accounted for by sex (~ 60%), Age (~ 6%), Race (~ 5%), and Height (3%) (Table 2). Blacks had a 12.4 (95% CI 5.4–19.4) mL/min/mmHg lower DLNO_5s_ compared to whites after controlling for sex, age, and height. Approximately 73% of the variance in DLCO_5s_ was accounted for by sex (~ 57%), race (10%), age (~ 4%), and height (2%) (Table 3). Blacks had a 3.9 (95% CI 2.5–5.3) mL/min/mmHg lower DLCO_5s_ compared to whites after controlling for sex, age, and height. Approximately 71% of the variance in VA_5s_ was accounted for by height (~ 60%), sex (~ 6%), and race (5%) (Table 4). Blacks had a 0.6 L (95% CI 0.4–0.9) L lower VA_5s_ compared to whites after controlling for sex, age, and height.

**Table 2:** Multiple linear regression results for DLNO (5 s breath-hold)

N = 118	Unstandardized coefficients		Standardized coefficients		95% Confidence interval for beta		Collinearity statistics	
	Beta	SE	Beta	*t*-statistic	Lower bound	Upper bound	Tolerance	VIF
Constant	− 1.69	39.67		− 0.043	− 80.3	76.9		
Sex (0 = females; 1 = males)	40.97	4.79	0.59	8.55*	31.5	50.5	0.52	1.91
Age^2^	− 0.013	0.002	− 0.27	− 5.27*	− 0.018	− 0.008	0.93	1.07
Race (0 = black; 1 = white	12.44	3.54	0.18	3.52*	5.4	19.4	0.95	1.05
Height (cm)	0.78	0.245	0.22	3.17*	0.3	1.3	0.52	1.92

	Sum of squares	Df	Mean square	F	Sig.			
*ANOVA results*								
Regression	103,381	4	25,845	74	0.000			
Residual	39,759	113	351.85					
Total	143,139	117						

^*^t-statistic was statistically significant (*p* < 0.01); SE = standard error; Durbin–Watson Statistic = 1.92; Overall R^2^ = 0.72; Standard error of the estimate (SEE) = 18.8 mL/min/mmHg. Sex, Age^2^, Race, and Height, accounted for approximately 60%, 6%, 5%, and 3% of the total variance in the model. ANOVA results were rounded to the nearest whole number except for Residual Mean Square error, which was rounded to two decimal places

**Table 3:** Multiple linear regression results for DLCO (5 s breath-hold)

N = 118	Unstandardized coefficients		Standardized coefficients		95% Confidence interval for beta		Collinearity statistics	
	Beta	SE	Beta	*t*-statistic	Lower bound	Upper bound	Tolerance	VIF
Constant	2.02	7.93		0.26	− 13.7	17.7		
Sex (0 = females; 1 = males)	8.40	0.96	0.60	8.77*	6.50	10.29	0.52	1.91
Race (0 = black; 1 = white	3.87	0.71	0.28	5.48*	2.5	5.3	0.95	1.05
Age^2^	− 0.002	0.00	− 0.23	− 4.44*	− 0.003	− 0.001	0.93	1.07
Height (cm)	0.14	0.049	0.19	2.78*	0.4	0.2	0.52	1.92

	Sum of squares	Df	Mean square	F	Sig.			
*ANOVA results*								
Regression	4209	4	1052	75	0.000			
Residual	1587	113	14.05					
Total	5796	117						

^*^t-statistic was statistically significant (*p* < 0.01); SE = standard error; Durbin–Watson Statistic = 2.08; Overall R^2^ = 0.73; Standard error of the estimate (SEE) = 3.8 mL/min/mmHg. Sex, Race, Age^2^, and Height, accounted for approximately 57%, 10%, 4%, and 2% of the total variance in the model. ANOVA results were rounded to the nearest whole number except for Residual Mean Square error, which was rounded to two decimal places

**Table 4:** Multiple linear regression results for alveolar volume (VA) (5 s breath-hold)

N = 118	Unstandardized coefficients		Standardized coefficients		95% Confidence interval for beta		Collinearity statistics	
	Beta	SE	Beta	*t*-statistic	Lower bound	Upper bound	Tolerance	VIF
Constant	− 6.16	1.41		− 4.36*	− 9.0	− 3.4		
Height (cm)	0.065	0.01	0.52	7.59*	0.05	0.08	0.55	1.83
Sex (0 = females; 1 = males)	0.835	0.17	0.34	5.02*	0.5	1.2	0.56	1.79
Race (0 = black; 1 = white	0.64	0.13	0.26	5.08*	0.39	0.90	0.96	1.04

	Sum of squares	Df	Mean square	F	Sig.			
*ANOVA results*								
Regression	129	4	43	95	0.000			
Residual	52	113	0.454		0.000			
Total	181	117						

^*^t-statistic was statistically significant (*p* < 0.01); SE = standard error; Durbin–Watson Statistic = 2.38; Overall R^2^ = 0.71; Standard error of the estimate (SEE) = 0.67 L. Height, Sex, and Race accounted for approximately 60%, 6%, and 5% of the total variance in the model. ANOVA results were rounded to the nearest whole number except for Residual Mean Square error, which was rounded to two decimal places

To confirm that there is a racial difference in DLNO_5s_ and to demonstrate that the results are like Table 2, we performed other analyses post-hoc. We decided to randomly sample a new set of 59 Caucasian subjects from the 2017 ERS Taskforce with similar heights and ages, and sex compared to the 59 African American subjects [27]. In this instance, approximately 66% of the variance in DLNO_5s_ was accounted for by sex (~ 51%), race (~ 9%), Age^2^ (~ 5%), Race (~ 9%), and Height (2%). This does suggest that race is an independent predictor of DLNO_5s_ between these age ranges.

## Discussion

This observational descriptive study ‘s main purpose was to evaluate racial differences in pulmonary diffusing capacity between black and white populations. As this was a pilot study, we collected data on ~ 60 young, healthy black adults and used those data to compare against a reference set of healthy white adults [27].

Our result demonstrates a small but important and significant racial difference in DLNO_5s_, DLCO_5s_, and VA_5s_. Approximately 5–10% of the total shared variance was accounted for by race in these main dependent variables. Interestingly, race was not a significant predictor for KNO_5s_ and KCO_5s_ (which is mathematically DLNO_5s_/VA_5s_ and DLCO_5s_/VA_5s_). This suggests that the lower VA_5s_ in blacks contributed to differences in DLNO_5s_ and DLCO_5s_. Total lung capacity was nearly 100% of predicted in the black population when prediction equations were applied for a white population [3], suggesting that there was additional dead space ventilation and/or increased residual volume in the black subjects.

A secondary aim was to compare differences in DLCO, VA and KCO in the black population at 10 s versus 5 s breath-holds. The DLCO was similar between both breath-hold times, however, VA was lower by about 150 mL when the 10 s breath-hold maneuver was performed (Additional file 1: Figures S4–S5). As such, KCO was about 0.16 mL/min/mmHg larger at 10 s vs 5 s breath-hold time. Similarly, Moinard and Guénard also demonstrated that VA was lower and KCO was larger at the longer breath-hold time in healthy subjects [62]. However, in their paper, these changes were non-significant due to the small sample size. Other studies show varying changes on DLCO, VA, and KCO at various breath-hold times in healthy subjects [39, 46, 63]. The conflicting data on varied breath-hold times affecting these parameters can be due to the heterogeneous ventilation distribution, or heterogeneous inspired gas penetration, between subjects in these studies. Independence of breath-hold time implies homogeneous distribution of KCO [64], and based on varied results between studies, some subjects demonstrated more heterogeneous ventilation distribution compared to other subjects, even though all subjects in these studies were classified as healthy [39, 46, 63].

Limited data exists on racial differences in DLNO. One previous study demonstrated that in sex, age, and height matched in white subjects, DLNO and DLCO were ~ 23, ~ 5 mL/min/mmHg higher, and VA was ~ 1 L more compared to African subjects [45]. That difference is higher compared to the 0.15 L difference identified in this study. Previous studies have also revealed that vital capacity was ~ 15% lower in blacks compared to age, height, and sex-matched whites [13, 43], and the DLCO was also lower in blacks compared to matched whites [13, 19]. Specifically, DLCO is lower by ~ 2 (~ 6%) mL/min/mmHg in black, age and height matched males compared with matched white males, and ~ 5 (~ 15%) mL/min/mmHg lower in black, age and height matched females compared to matched white females, respectively [19]. Thus, the difference in DLCO between blacks and whites stemmed from differences in VA and hemoglobin concentration as there was a ~ 6% larger hemoglobin concentration in whites compared to blacks [19].

This study revealed, when matched for age and height, DLNO is ~ 8% lower in African American males and ~ 10% lower in African American females compared to whites. If 2.5% of this population is considered to have abnormally low DLNO,^1^ then approximately 862,000 African Americans ≥ 18 years of age in the United States could theoretically have lung disease.^2^ If a prediction equation created for the white population was applied to blacks, lung disease would be over diagnosed by approximately 8%, with significant implications for approximately 2.65 million individuals. This false positive misdiagnosis could increase patient stress and the inadvertent use of healthcare resources could result in a higher cost for a non-illness. Currently, there is no evidence that having a high DLNO is considered a pathologic state; thus underestimating the upper limit of normal for a black population when using a prediction equation for whites has no real consequence.

On the other hand, having a large DLCO, in certain cases, can suggest a pathological state. For example, those who are diagnosed with Goodpasture’s Syndrome [65], or who are obese [66], polycythemic [67], or those with a pulmonary hemorrhage [68], can have an abnormally high DLCO. Thus, depending on the prevalence of each of these conditions in the black population, applying the wrong reference equation can result in additional harm. The consequences of providing a false negative to a patient for DLCO could significantly further impact the overall cost of healthcare, as well as negatively impact survival, and provide false reassurance. [69]. A false-negative result is a missed opportunity, and may require more aggressive treatment down the line with potential medical legal consequences as well. [69].

What about comparing the false-negative rate using the traditional DLCO test with that of the relatively new DLNO test irrespective of race? Recent data has demonstrated that the false-negative rate for diagnosing various cardiopulmonary diseases after measuring DLNO was 10%, but for DLCO, it was 16% [26]. Thus, there was a 6% lower false-negative rate when using DLNO as a diagnostic test compared to DLCO.

Our study benefited from the technical quality of the PFTs, further supporting our conclusions (Additional file 1: Tables S4, S5, S7). Mean inspired volumes were 94% of the FVC during the DLCO_10s_ and 89% of the FVC for the DLNO_5s_ test. Breath-hold time varied between 8 and 12 s for the 10 s breath hold test and 4–6 s for the 5 s breath-hold test. The mean VA_5s_ and VA_10s_ was 91% and 88% of the measured TLC from the body plethysmograph, respectively.

There are a few caveats to consider. First, data shows that fitness may impact DLNO_5s_ and DLCO_5s_ [70, 71]. Although this may be true; however, the Global Lung Function Initiative (GLI) equations for pulmonary diffusing capacity (the gold standard) also do not account for fitness [8]. Fitness may also be a factor in racial differences of DLNO. However, this does not negate the need for racially appropriate reference equations. It would not be feasible to measure aerobic capacity in every patient prior to a pulmonary function test. Despite this suggestion, it has been shown that the mean difference in aerobic capacity between blacks and whites is 2.5 mL/kg/min [72], which is too small to cause a difference in pulmonary diffusing capacity. For example, every 1 mL/kg/min increase in aerobic capacity, DLNO_5s_ increases by about 1 mL/min/mmHg [70]. Thus, a ~ 2.5 mL/kg/min lower aerobic capacity in blacks should result in a ~ 2.5 and not a 12 mL/min/mmHg lower DLNO_5s_ in blacks, as we demonstrated. Another caveat to consider is that we used a fixed hemoglobin concentration as it was not measured. However, we believe that this does not diminish our findings. First, in major hospital labs, measuring hemoglobin concentration prior to every pulmonary function test is not practical. Second, the Global Lung Function Initiative reference equations for DLCO_10s_ do not adjust for hemoglobin concentration [8] (see explanation on the bottom of page 8 and the top of page nine in ERS technical standards of why this is not necessary to correct for Hb [8]). In essence, correcting for hemoglobin does not improve overall model fit [8]. Third, in our previous work, we also demonstrate minimal differences in DLCO when using corrected vs. non-corrected hemoglobin values [30]. Fourth, DLNO is minimally affected by Hb [29]. Thus, DLCO and DLNO hemoglobin correction is not needed. Finally, a recent article published in 2021 demonstrates that the Hyp'Air pulmonary function device measures DLNO that may be about 17% higher than that of the Masterscreen PFT Pro  equipment (Carefusion, USA) [73]).  These between machine differences could confound the findings of this study. However, the between machine differences are unlikely to affect the current findings since the reference equations published in the ERS technical standards [27] are mostly from subjects that had DLNO assessed by the Hyp'Air device, which is the same device used in this study.  We did not use the Masterscreen PFT system for the measurement of DLNO. 

## Conclusion

In conclusion, the results of this pilot data reveal small but important and statistically significant racial differences in DLNO and DLCO in adults, which are due, in part, to differences in VA. Future reference equations should account for racial differences. If these differences are not accounted for, then the risk of falsely diagnosing lung disease increase in blacks when using reference equations for whites.

## Supplementary Information

**Additional file 1.** Supplementary figures and tables.

## Data Availability

The datasets used and/or analyzed during the current study are available from the corresponding author [GSZ] on reasonable request.

## Abbreviations

ATS: American Thoracic Society
CO: Carbon monoxide
COHb: Carboxyhemoglobin
COPD: Chronic obstructive pulmonary disease
DL: Diffusing capacity of the lung
DLCO: Diffusing capacity of the lung for carbon monoxide (mL/min/mmHg)
DLNO: Diffusing capacity of the lung for nitric oxide (mL/min/mmHg)
DMCO: Alveolar-capillary membrane diffusing capacity for CO
ERS: European Respiratory Society
FEV_1_: Forced expiratory volume in 1 s
FVC: Forced vital capacity
FRC: Functional residual capacity
GSU: Georgia State University
Hb: Hemoglobin
KCO: Logarithmic change in CO concentration per unit time and unit pressure (mL STPD/min/mmHg/L) and mathematically equivalent to DLCO divided by alveolar volume
NO: Nitric oxide
KNO: Logarithmic change in NO concentration per unit time and unit pressure mL (STPD/min/mmHg/L) and mathematically equivalent to DLNO divided by alveolar volume
LLN: Lower limit of normal, taken as the 2.5th percentile
PFTs: Pulmonary function tests
VA: Alveolar volume (L)
Vc: Pulmonary capillary blood volume (mL)
TLC: Total lung capacity (L)
ULN: Upper limit of normal, taken as the 97.5th percentile

## Footnotes

1 A z-score of -1.96 SD units (2.5th percentile) is considered the LLN for case finding purposes of asymptomatic subjects based on a standard Gaussian distribution (Eur Respir J. 2012;40(6):1324–43).

2 As of 2021, there are about 34,506,788 African Americans in the U.S ≥ 18 years of age.
