# Supplementary material for: The need for race-specific reference equations for pulmonary diffusing capacity for nitric oxide
Source: BMC Pulm Med. 2021 Jul 13;21:232. doi: 10.1186/s12890-021-01591-7 (PMC8278768; doi:10.1186/s12890-021-01591-7)
Supplement: Supplementary file 1 — Additional file 1. Supplementary figures and tables. [file 12890_2021_1591_MOESM1_ESM.docx]

#

**The need for race-specific reference equations for pulmonary diffusing capacity for nitric oxide**

# Supplementary List of Tables and Figures

^1^Gerald Stanley Zavorsky PhD, RCP, RPFT, FACSM; ^2^Ahmad Saleh Almamary MS; ^3^Mobarak Khalid Alqahtani MS; ^4^Shi Huh Samuel Shan MS, RRT-NPS, ACCS; ^4^Douglas Shawn Gardenhire, EdD, RRT-NPS, FAARC

^1^Pulmonary Services Department, University of California, Davis, Medical Center, Sacramento, CA

^2^ Faculty of Medicine, National Heart and Lung Institute, Imperial College London, London, UK

^3^Institute of Cardiovascular Sciences, University of Birmingham, Birmingham, UK

^4^Department of Respiratory Therapy, Georgia State University, Atlanta, GA

## Table S1**.** DLNO prediction equations in the scientific literature. These studies used the double diffusion NO-CO technique.

| **Study** | **Prediction equation for DLNO_5s_** | **BHT** | **Number of subjects** | **R^2^** | **SEE** |
| --- | --- | --- | --- | --- | --- |
| **Adults (White subjects)** |  |  |  |  |  |
| van der Lee *et al*. (2007)[1]. Females (mmoL/min/kPa) | 0.535·(height in cm) – 0.077·(age) – 48.28 | 10 s | 59 F | -- | 5.2 |
| van der Lee *et al.* (2007)[1]. Males (mmoL/min/kPa) | 0.598·(height in cm) – 0.25·(age) – 44.2 | 10 s | 65 M | -- | 6.4 |
| Aguilaniu *et al.* (2008)[2].  (mL/min/mmHg)  >59 years old | 137·(height in cm) – 0.90·(age)+27.35·(Sex) – 54.69 | 4 s | 59 F  45 M | 0.72 | -- |
| Aguilaniu *et al.* (2008)[2].  (mL/min/mmHg)  ≤ 59 years old | 136.5·(height in cm) – 0.88 · (age)+27.35·(Sex) – 54.69 | 4 s | 59 F  45 M | 0.72 | -- |
| Zavorsky *et al.* (2008)[3].  (mL/min/mmHg) | 1.17·(height in cm) – 1.21 · (age) + 31.81·(Sex) – 20.1 | 5 s | 64 F  66 M | 0.70 | 20.2 |
| Zavorsky *et al.* (2017)[4].  (mL/min/mmHg) | 0.81·(height in cm) – 0.010·(age^2^) + 34.4·(sex)+ 9.7 | 6 s | 242 F  248 M | 0.69 | 20.0 |
| Munkholm *et al.* (2018) [5]. Females (mL/min/mmHg) | 0.766∙(height in cm) – 0.00753∙(age^2^) – 2.36 | 6 s | 142 F | 0.80 | 11.4 |
| Munkholm *et al.* (2018) [5]. Males (mL/min/mmHg) | 0.97∙(height in cm) – 0.0125∙(age^2^) + 5.72 | 6 s | 138 M | 0.82 | 16.6 |
| **Children** |  |  |  |  |  |
| Thomas *et al.* (2014)[6].  (mmoL/min/kPa)  (White subjects) | Exp [1.3145 + 0.0214·(age) – 0.0058·(sex) + 0.0119· (height in cm) – 1.2893·10^-8^ · (height in cm)^3^ + 2.7070·10^-8^·(Sex) · (height in cm)^3^] | 5 s | 312 | -- | -- |
| Dridi *et al.* (2020)[7].  (mL/min/mmHg)  (Tunisian Boys) | 0.76∙(height in cm) – 24.4 | 4 s | 118 | 0.70 | -- |

BHT = breath-hold time (s); SEE = standard error of the estimate; Sex (1 = male, 0 = female). Note: There were prediction equations developed by Rouatbi *et al.* (2010) for North-African adults where DmCO and Vc were estimated based on DLCO and DLNO [8]. However, no actual prediction equations for DLNO were found in that article. As such, Rouatbi’s paper using for North-African adults is not included in this table.

Table S2. DLCO prediction equations in the scientific literature. These studies used the double diffusion NO-CO technique.

| **Study** | **Prediction equation for DLCO_5s_** | **BHT** | **Number of subjects** | **R^2^** | **SEE** |
| --- | --- | --- | --- | --- | --- |
| **Adults (White subjects)** |  |  |  |  |  |
| van der lee *et al.* (2007)[1]. Females (mmoL/min/kPa) | 10.51· (height in m) – 0.030·(age) – 7.43 | 10s | 59 F | -- | 1.4 |
| van der lee *et al.* (2007)[1].  Males (mmoL/min/kPa) | 12.02· (height in m) –0.074·(age) – 6.88 | 10s | 65 M | -- | 1.7 |
| Aguilaniu *et al.* (2008)[2].  (mL/min/mmHg)  >59 years old | 29.29·(height in m) – 0.26·(age) + 5.044·(Sex) – 12.95 | 4 s | 59 F  45 M | 0.73 | -- |
| Aguilaniu *et al.* (2008)[2].  (mL/min/mmHg)  ≤ 59 years old | 29.29· (height in m) – 0.16·(age) + 5.044·(Sex) – 12.95 | 4 s | 59 F  45 M | 0.73 | -- |
| Zavorsky *et al.* (2008)[3].  (mL/min/mmHg) | 0.214· (height in cm) – 0.242 · (age) + 5.94·(Sex) – 1.3 | 5s | 64 F  66 M | 0.69 | 4.0 |
| Zavorsky *et al.* (2017)[4]  Males (mL/min/mmHg) | 0.23· (height in cm) – 0.002·(age^2^) + 6.0 · (sex) – 8.5 | 6s | 242 F  248 M | 0.68 | 4.2 |
| Munkholm *et al.* (2018)[5]  Females (mL/min/mmHg) | 0.192∙(height in cm) – 0.00166 ∙ (age^2^) – 3.58 | 6 s | 141 F | 0.77 | 2.8 |
| Munkholm *et al.* (2018) [5]  Males (mL/min/mmHg) | 0.252∙(height in cm) – 0.00258∙ (age^2^) – 5.01 | 6 s | 139 M | 0.81 | 3.7 |
| **Children** |  |  |  |  |  |
| Thomas *et al.* (2014) [6]. (mmoL/min/kPa)  (White subjects) | Exp [0.9440 + 0.0205·(age) + 0.0908·(sex) + 1.6233·10^-7^ · (height in cm)^-3^] | 5 s | 312 | -- | -- |
| Dridi *et al.* (2020)[7].  mL/min/mmHg)  (Tunisian Boys) | 0.20∙(weight in kg) + 8.98 | 4 s | 118 | 0.64 | -- |

BHT – breath-hold time (s); Sex (1 = male, 0 = female). Note: There were prediction equations developed by Rouatbi et al. (2014) for North-African children where DmCO and Vc were estimated based on DLCO and DLNO [9]. However, no actual prediction equations for DLNO were found in that article. As such, Rouatbi’s paper using North African children is not included in this table.

Table S3. More detailed anthropometric characteristics of the African American subjects

|  | **Males (n = 27)** | **Females**  **(n = 32)** | **Combined (n = 59)** |
| --- | --- | --- | --- |
| **Age (years)** | 28 (10)  [18 to 55] | 32 (14)  [20 to 67] | 30 (12)  [18 to 67] |
| **Weight (kg)** | 78.6 (11.1)  [61 to 102.4] | 68.3 (13.9)  [47.8 to 95.7] | 73.0 (13.6)  [47.8 to 102.4] |
| **Height (cm)** | 176.2 (6.7)  [163.1 to 189.4] | 163.2 (6.7)  [140.2 to 180.0] | 169 (10)  [140 to 189] |
| **Body mass index (kg/m^2^)** | 25.3 (2.9)  [18.8 to 30.6] | 25.3 (4.4)  [17.2 to 32.3] | 25.3 (3.8)  [17.2 to 32.3] |
| **Waist Circumference (cm)** | 83.7 (8)  [66 to 100] | 82.3 (11)  [65 to105] | 82.9 (9.7)  [65 to 105] |
| **Hip Circumference (cm)** | 101.8 (6.9)  [90.5 to 119.0] | 102.5 (9.6)  [87.6 to 119.0] | 102.2 (8.4)  [87.6 to 119] |
| **WHR** | 0.82 (0.04)  [0.72 to 0.90] | 0.80 (0.06)  [0.69 to 0.96] | 0.81 (0.05)  [0.69 to 0.96] |

Mean (SD). Brackets represent the range. Body mass index (BMI) is calculated weight (kg)

divided by height^2^ (meters). WHR = waist-to-hip ratio

## Table S4. Spirometric results of the African American subjects

|  | **Mean value** | **% predicted** | ***p*-value** |
| --- | --- | --- | --- |
| **SVC (L)** | 4.16 (0.94) | -- | -- |
| **FVC (L)** | 4.20 (0.91) | 111 (11) | 0.000 |
| **FEV_1_ (L)** | 3.48 (0.72) | 109 (13) | 0.000 |
| **FEV_1_/FVC** | 0.83 (0.07) | 98 (7) | 0.014 |
| **TLC (L)** | 5.82 (1.24) | 99 (11) | 0.529 |

5 of 59 African American subjects (~8%) demonstrated a mild obstructive pattern based on being below the LLN for FEV_1_/FVC. The % predicted for FVC, FEV_1_, and FEV_1_/FVC was based on the Global Lung Function Initiative prediction equations for blacks [10]. The % predicted TLC was from white prediction equations from Verbanck and colleagues [11].

## Table S5. The differences in DLCO between the two different pieces of equipment which used two different breath-hold times.

|  | **Body Plethysmograph (10s BHT)** | **Hyp’Air**  **(5s BHT)** | **∆** | ***p* value** | |
| --- | --- | --- | --- | --- | --- |
| **VA (L)** | 5.1 (1.1) | 5.3 (1.2) | -0.15 (0.40) | | 0.004 |
| **DLCO (mL/min/mmHg)** | 26.6 (6.4) | 26.6 (7.0) | -0.1 (3.1) | | 0.868 |
| **Inspired volume (L)** | 3.95 (0.86) | 3.72 (0.84) | 0.24 (0.28) | | 0.000 |

Mean (SD). BHT = breath-hold time. The Body Plethysmograph used was the BODYBOX 5500^®^ Series (Medisoft Inc., Sorinnes, Belgium). The Hyp’Air lung diffusion system was also from the same company. However, the software used by the body plethysmograph was the ComPAS software, developed by Morgan Scientific (Haverhill, MA), whereas in the Hyp’Air lung diffusion system, it was Exp’air Software.

## Table S6. Pulmonary diffusing capacity results in African American subjects.

|  | **Males**  **(n = 27)** | **Females**  **(n = 32)** | **Combined**  **(n = 59)** |
| --- | --- | --- | --- |
| **Hyp’Air (5 s BHT)** | | | |
| **DLNO (mL/min/mmHg)** | 165 (23)  [120 to 205] | 110 (162)  [71 to 138] | 135 (34)  [71 to 205] |
| **DLCO (mL/min/mmHg)** | 32.9 (5.0)  [23.1 to 43.9] | 21.4 (2.6)  [16.6 to 27.0] | 26.6 (7)  [16.6 to 43.9] |
| **VA (L)** | 6.2 (0.9)  [5.8 to 7.9] | 4.5 (0.7)  [3.1 to 6.3] | 5.3 (1.2)  [3.1 to 7.9] |
| **KCO (mL/min/mmHg/L)** | 5.3 (0.8)  [3.5 to 6.7] | 4.8 (0.6)  [3.6 to 6.0] | 5.0 (0.8)  [3.5 to 6.7] |
| **KNO (mL/min/mmHg/L)** | 26.7 (3.8)  [18.6 to 32.9] | 24.6 (3.3)  [18.3 to 29.6] | 25.5 (3.6)  [18.3 to 32.9] |
| **DLNO/DLCO ratio** | 5.0 (0.3)  [4.3 to 5.5] | 5.1 (0.4)  [4.3 to 6.3] | 5.1 (0.4)  [4.3 to 6.3] |
| **Body Plethysmograph (10 s BHT)** | | | |
| **DLCO (mL/min/mmHg)** | 32.6 (4.0)  [26.6 to 44.6] | 21.5 (2.3)  [16.4 to 26.8] | 26.6 (6.4)  [16.4 to 44.6] |
| **VA (L)** | 6.0 (0.8)  [4.5 to 8.4] | 4.4 (0.7)  [3.1 to 5.9] | 5.1 (1.1)  [3.1 to 8.4] |
| **KCO (mL/min/mmHg/L)** | 5.5 (0.8)  [3.4 to 7.4] | 5.0 (0.8)  [3.4 to 6.8] | 5.2 (0.8)  [3.4 to 7.4] |
| **TLC (L)** | 6.8 (1.0)  [5.0 to 9.3] | 5.0 (0.7)  [4.0 to 7.0] | 5.8 (1.2)  [4.0 to 9.3] |

Mean (SD). Brackets represent the range. BHT = breath-hold time

## Table S7. Inhaled and exhaled gases including breath-hold time (BHT) for both PFT machines in African American subjects (Hyp’Air and Body Plethysmograph).

|  | **O_2_** (%) | | **CO (%)** | | **He (%)** | | **NO (%)** | | **BHT** |
| --- | --- | --- | --- | --- | --- | --- | --- | --- | --- |
|  | ***Inspire*** | ***Expire*** | ***Inspire*** | ***Expire*** | ***Inspire*** | ***Expire*** | ***Inspire*** | ***Expire*** |  |
| **Hyp’Air** | 19.7 (0.12) | 16.8 (0.54) | 0.30 (0.00) | 0.10 (0.02) | 9.5 (0.6) | 6.0 (0.6) | 43 (4) | 4 (1.0) | 5.6  (0.6) |
| **Body Plethysmograph** | 20.7 (2.4) | 17.7 (0.6) | 0.30 (0.01) | 0.10 (0.01) | 10 (0.1) | 7.0 (0.6) | -- | -- | 10.3  (0.5) |

Mean (SD). BHT = breath-hold time. The Body Plethysmograph used was the BODYBOX 5500^®^ Series (Medisoft Inc., Sorinnes, Belgium). The Hyp’Air lung diffusion system was also from the same company. However, the software used by the body plethysmograph was the ComPAS software, developed by Morgan Scientific (Haverhill, MA), whereas in the Hyp’Air lung diffusion system, it was Exp’air Software.

## Figure S1. The association between DLCO_10s_ and DLCO_5s_ in black subjects (n = 59). There is an ~80% shared variance between the two variables. The equation is DLCO_10s_ = 0.83∙(DLCO_5s_) + 4.54. SEE = 2.94 mL/min/mmHg, *p* < 0.001. The coefficient of variation was ~8% between the DLCO values measured at the two different breath-hold times.

| 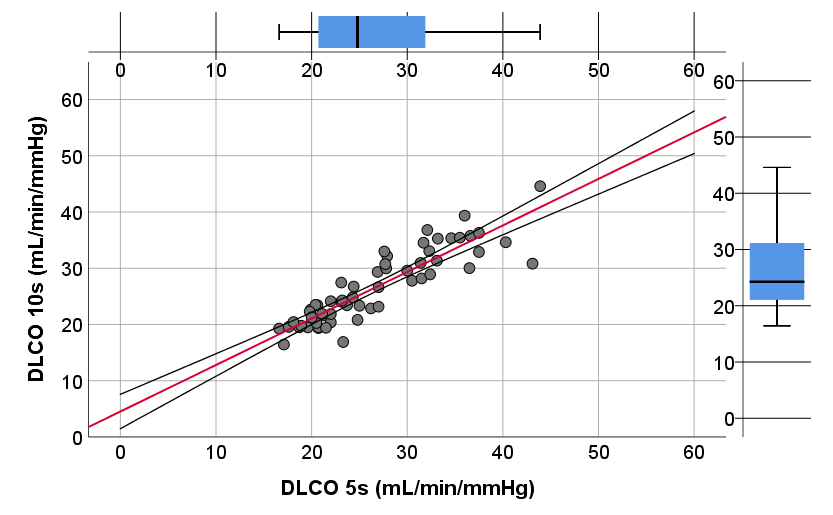 |
| --- |

Figure S2. The association between VA_10s_ and VA_5s_ in black subjects (n = 59). There is an ~88% shared variance between the two variables. The equation is VA_10s_ = 0.91∙(VA_5s_) + 0.34. SEE = 0.39 L, *p* < 0.001. The coefficient of variation was ~6% between the VA values measured at the two different breath-hold times.

| 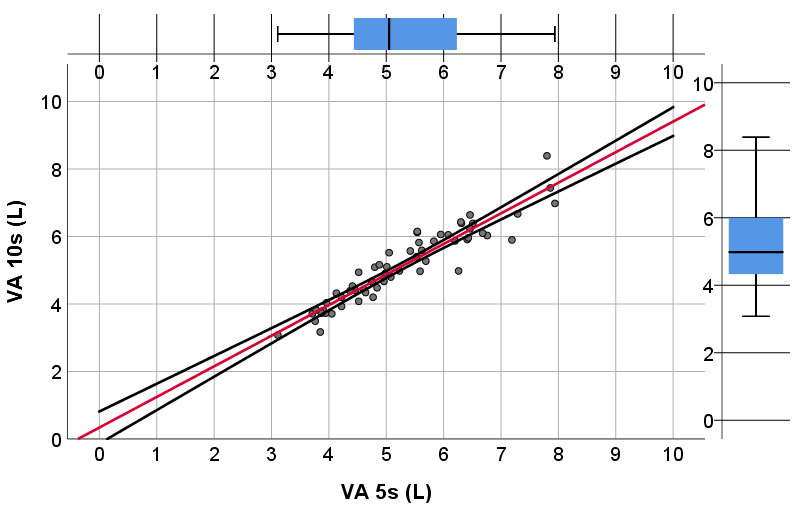 |
| --- |

Figure S3. The association between KCO_10s_ and KCO_s_ in black subjects (n = 59). There is an ~74% shared variance between the two variables. The equation is KCO_10s_ = 0.91∙(KCO_5s_) + 0.61. SEE = 0.42 mL/min/mmHg/L, *p* < 0.001. The coefficient of variation was ~6% between the KCO values measured at the two different breath-hold times.

| 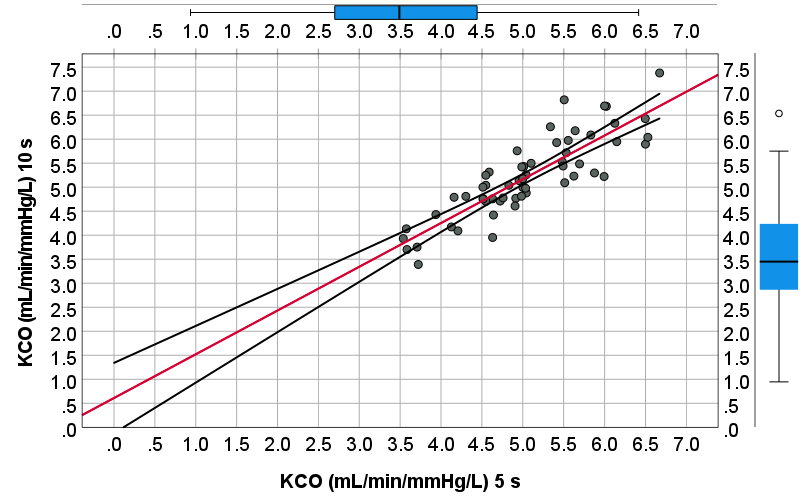 |
| --- |

Figure S4. A Bland-Alman plot showing the differences in DLCO_10s_ and DLCO_5s_ in black subjects (n = 59). The mean difference was 0.1 mL/min/mmHg (SD of the difference = 3.1 mL/min/mmHg). No statistical difference between the two measurements (paired *t*-test, *p* = 0.88).

| 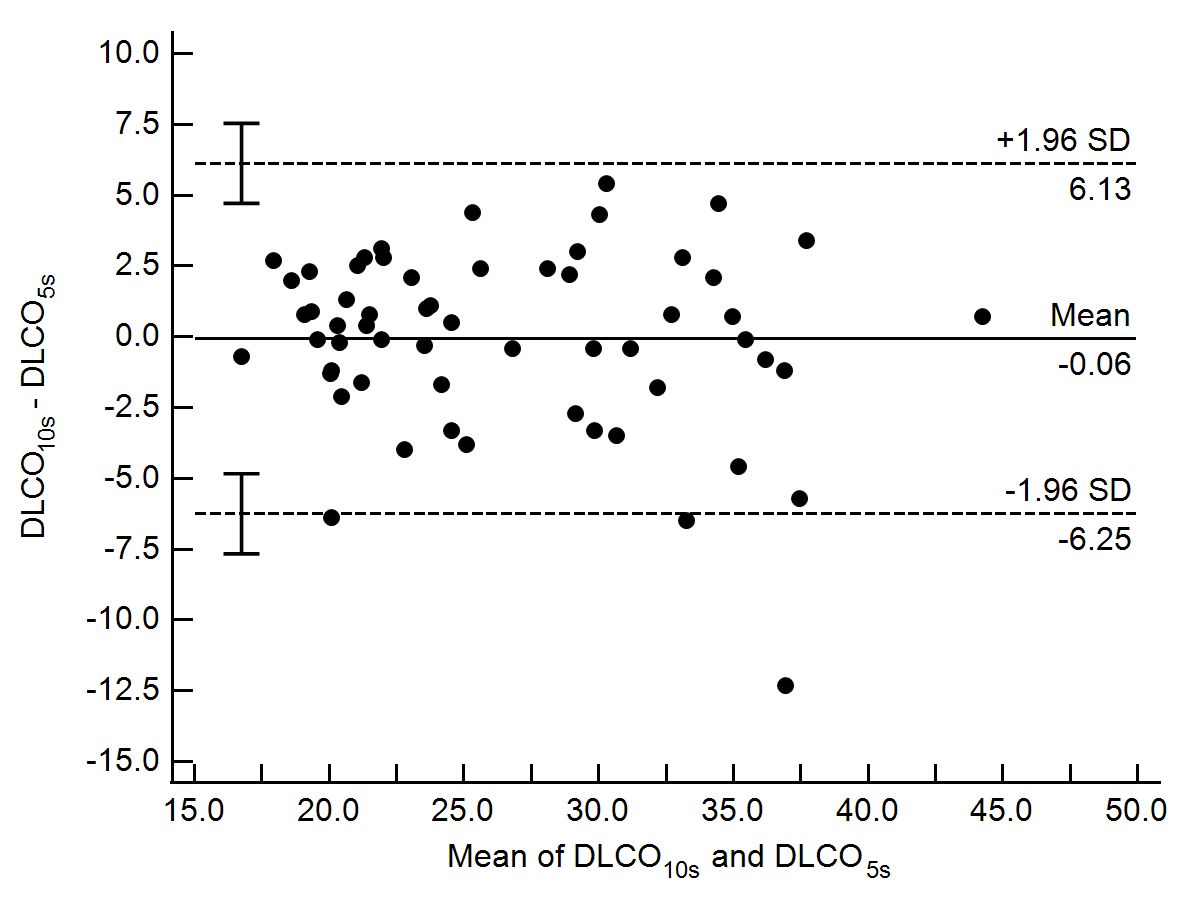 |
| --- |

Figure S5. A Bland-Alman plot showing the differences in VA_10s_ and VA_5s_ in black subjects (n = 59). The mean difference was 0.15 L (SD of the difference = 0.40 L). There was statistical difference between the two measurements (paired *t*-test, p = 0.004).

| 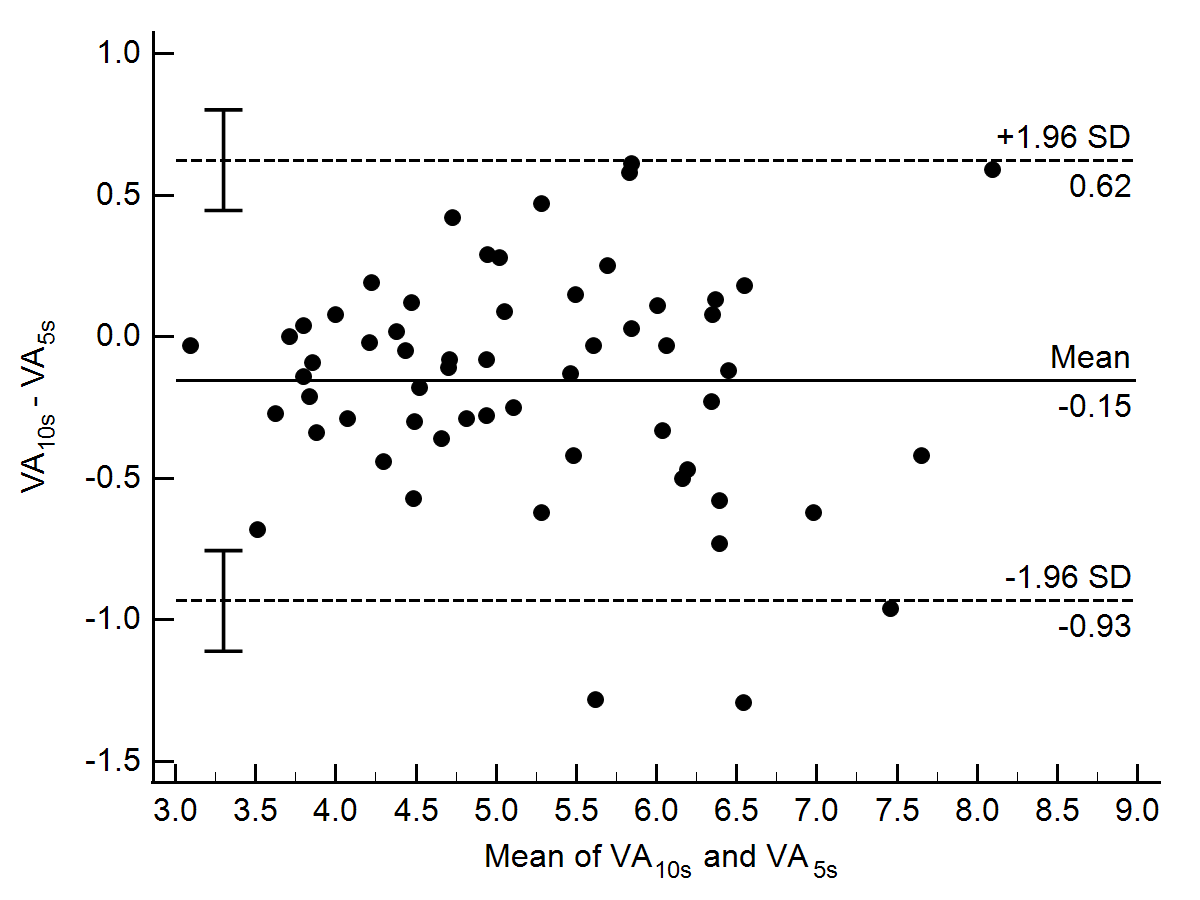 |
| --- |

Figure S6. A Bland-Alman plot showing the differences KCO_10s_ and KCO_5s_ in black subjects (n = 59). The mean difference was 0.16 mL/min/mmHg/L) (SD of the difference = 0.43 mL/min/mmHg/L). There was statistical difference between the two measurements (paired *t*-test, *p* = 0.005)

| 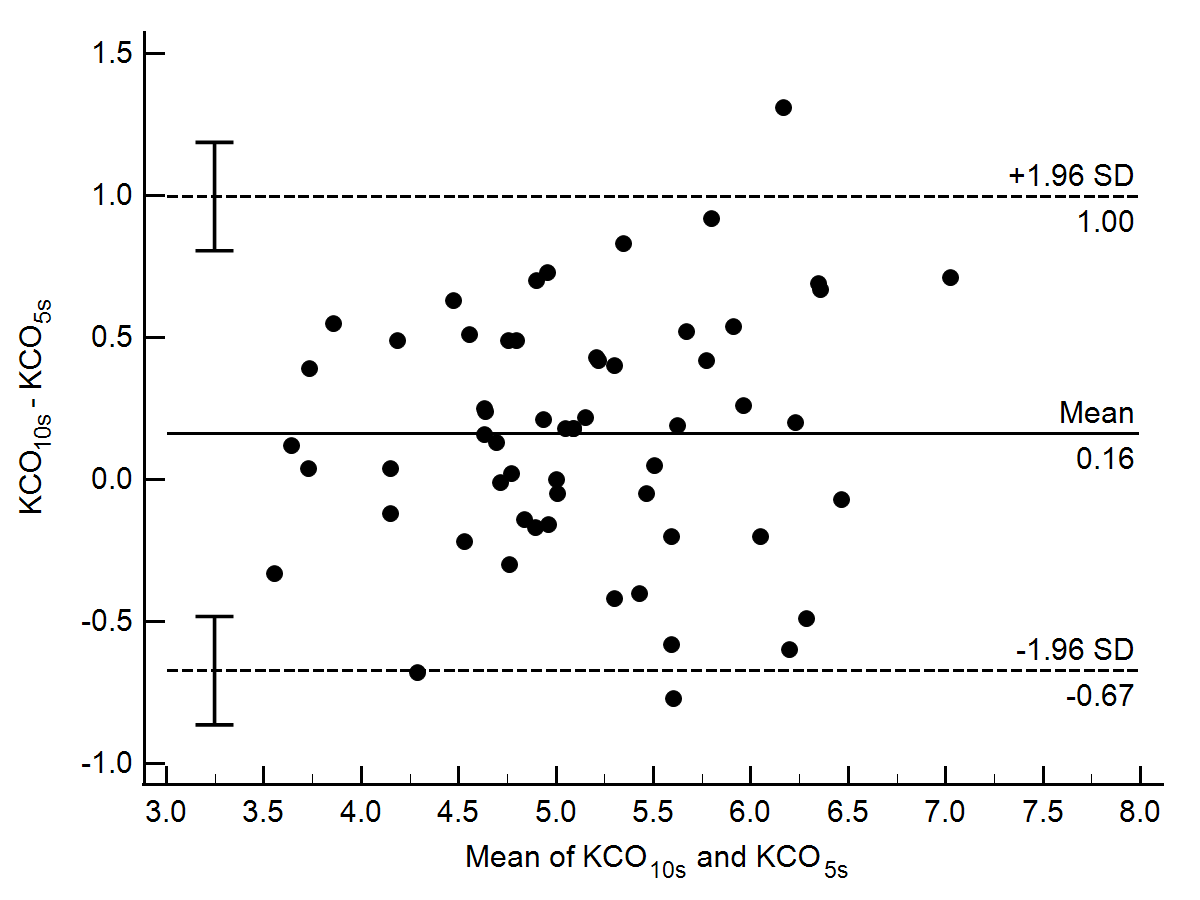 |
| --- |

## Figure S7. The association between resting heart rate (HR) and pulmonary diffusing capacity in African American Adults from the double diffusion NO-CO technique. The figures show that there is only a 8-12% shared variance between diffusing capacity and heart rate at rest. However, when controlling for age, the percentage of shared variance increased, to 18-21%.

| 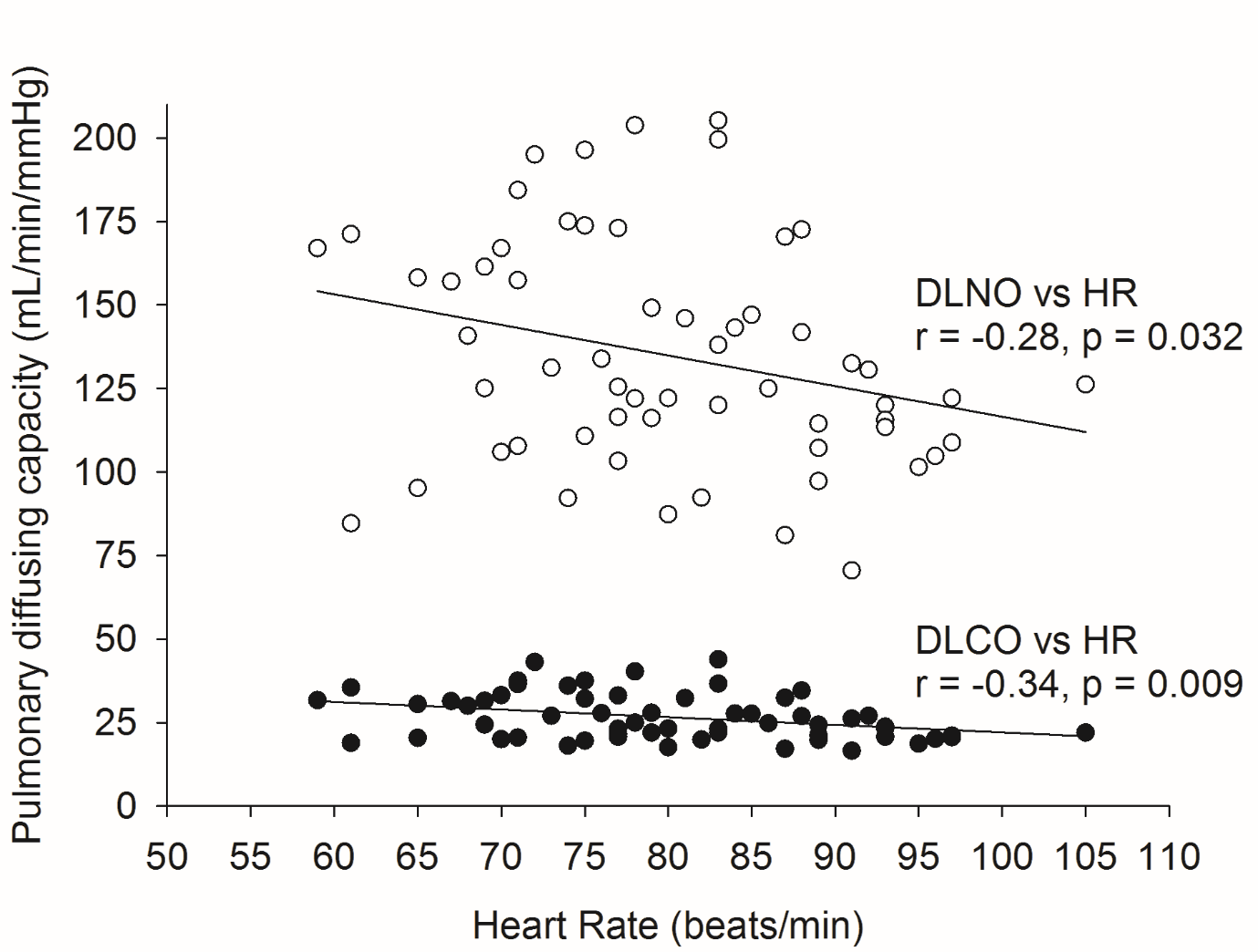 |
| --- |

**References**

1. van der Lee I, Zanen P, Stigter N, van den Bosch JM, Lammers JW. Diffusing capacity for nitric oxide: reference values and dependence on alveolar volume. Respir Med. 2007;101(7):1579-84.

2. Aguilaniu B, Maitre J, Glenet S, Gegout-Petit A, Guenard H. European reference equations for CO and NO lung transfer. Eur Respir J. 2008;31(5):1091-7.

3. Zavorsky GS, Cao J, Murias JM. Reference values of pulmonary diffusing capacity for nitric oxide in an adult population. Nitric Oxide. 2008;18(1):70-9.

4. Zavorsky GS, Hsia CC, Hughes JM, Borland CD, Guenard H, van der Lee I, et al. Standardisation and application of the single-breath determination of nitric oxide uptake in the lung. Eur Respir J. 2017;49(2):1600962.

5. Munkholm M, Marott JL, Bjerre-Kristensen L, Madsen F, Pedersen OF, Lange P, et al. Reference equations for pulmonary diffusing capacity of carbon monoxide and nitric oxide in adult Caucasians. Eur Respir J. 2018;52(1): 1500677.

6. Thomas A, Hanel B, Marott JL, Buchvald F, Mortensen J, Nielsen KG. The single-breath diffusing capacity of CO and NO in healthy children of European descent. PLoS One. 2014;9(12):e113177.

7. Dridi R, Dridi N, Ben Moussa Zouita A, Muller PT, Tabka Z, Guenard H, et al. Pulmonary diffusing capacity measured by NO/CO transfer in Tunisian boys. Pediatr Pulmonol. 2020;55(10):2754-61.

8. Rouatbi S, Ben Saad H, Latiri I, Tabka Z, Guenard H. North-African reference values of alveolar membrane diffusion capacity and pulmonary capillary blood volume. Respiration. 2010;80(4):301-12.

9. Rouatbi S, Khemis M, Garrouche A, Saad HB. Reference values of capillary blood volume and pulmonary membrane diffusing capacity in North African boys aged 8 to 16 years. Egypt J Chest Dis Tuber. 2014;63(3):705-15.

10. Quanjer PH, Stanojevic S, Cole TJ, Baur X, Hall GL, Culver BH, et al. Multi-ethnic reference values for spirometry for the 3-95-yr age range: the global lung function 2012 equations. Eur Respir J. 2012;40(6):1324-43.

11. Verbanck S, Van Muylem A, Schuermans D, Bautmans I, Thompson B, Vincken W. Transfer factor, lung volumes, resistance and ventilation distribution in healthy adults. Eur Respir J. 2016;47(1):166-76.
